# Landscape Profiling Analysis of DPP4 in Malignancies: Therapeutic Implication for Tumor Patients With Coronavirus Disease 2019

**DOI:** 10.3389/fonc.2021.624899

**Published:** 2021-02-04

**Authors:** Lingling Shu, Yang Liu, Jinyuan Li, Xiaoping Wu, Yang Li, Hanying Huang

**Affiliations:** ^1^ State Key Laboratory of Oncology in South China, Collaborative Innovation Center for Cancer Medicine, Sun Yat-sen University Cancer Center, Guangzhou, China; ^2^ Department of Hematological Oncology, Sun Yat-sen University Cancer Center, Guangzhou, China; ^3^ Key Laboratory of Neurosurgery in Guangdong Province, Southern Medical University, Guangzhou, China; ^4^ Department of Neurosurgery, Zhujiang Hospital, Southern Medical University, Guangzhou, China; ^5^ State Key Laboratory of Pharmaceutical Biotechnology, The University of Hong Kong, Hong Kong, Hong Kong

**Keywords:** DPP4, malignancies, immunity, SARS-CoV-2, bioinformatics

## Abstract

Severe coronavirus disease 2019 (COVID-19), caused by severe acute respiratory syndrome coronavirus 2 (SARS-CoV-2), is characterized by pneumonia, lymphopenia, and cytokine storms. Patients with underlying conditions, and especially cancer patients with impaired immunity, are particularly vulnerable to SARS-CoV-2 infection and complications. Although angiotensin converting enzyme II (ACE2) has been identified as a cellular binding receptor for SARS-CoV-2, immunopathological changes in severe cancer patients support the investigation of additional potential receptors such as dipeptidyl peptidase 4 (DPP4), a key immunoregulator. However, a comprehensive profiling analysis of DPP4 in malignancies remains obscure. In this study, using different datasets, we demonstrated the expression of DPP4 in healthy tissues and pan-cancers, showing the risk of different cancer types towards SARS-CoV-2 infection according to DPP4 expression levels. DPP4 expression was positively correlated with infiltrating levels of various immune cells and showed strong correlations with diverse immune marker sets in pan-cancer patients analyzed by Tumor Immune Estimation Resource (TIMER). These findings suggest that increased DPP4 expression in specific cancer patients might account for the high susceptibility to SARS-CoV-2 infection and the induction of cytokine storms. Due to the critical role of DPP4 in immunometabolism, our results indicate that pharmacological inhibition of DPP4 might provide beneficial therapeutic effects for SARS-CoV-2 treatment together with other strategies in specific tumor patients.

## Introduction

Coronavirus disease 2019 (COVID-19), a newly emerged respiratory disease caused by severe acute respiratory syndrome coronavirus 2 (SARS-CoV-2), has become a worldwide pandemic with 21,294,845 cases and 761,779 deaths as of August 16, 2020 (1). Although most patients infected with SARS-CoV-2 exhibit mild to moderate symptoms, approximately 15% progress to severe pneumonia, and approximately 5% eventually develop acute respiratory distress syndrome (ARDS) and multiple organ dysfunction (2). At present, the mortality rate of COVID-19 worldwide is approximately 2.4%, which is caused by multi-organ failure, especially in elderly people and people with underlying health conditions such as hypertension, cardiovascular disease, and diabetes (3). Cancer patients with defective immunity are especially susceptible to SARS-CoV-2 infection (4). Furthermore, the so-called cytokine storm, characterized by high levels of various proinflammatory cytokines, including IL-2, IL-7, IL-10, CXCL10 (IP-10), MCP-1(CCL2), and TNFα, has been observed in severe cases of COVID-19 (2), indicating that immune regulation has a critical role in the pathogenesis of COVID-19 (5, 6).

Numerous studies have shown that, similar to SARS-CoV, SARS-CoV-2 requires angiotensin converting enzyme 2 (ACE2) as a receptor to enter cells (7). It has also been shown that obese or specific cancer patients are more susceptible to SARS-CoV-2 infection, mainly due to the high expression of ACE2 (8, 9). The binding of the virus to host cell receptors is a significant determinant of the pathogenesis of infection. In addition to ACE2, there are several additional potential receptors for SARS-CoV-2. Dipeptidyl peptidase 4 (DPP4, also known as CD26), is the third exo-peptidase found to be a coronavirus receptor (after ACE2 and aminopeptidase, APN) (10). DPP4 is a multifunctional type II transmembrane glycoprotein with 766 amino acids and exists as a homodimer on the cell surface; it belongs to the family of proline-specific serine proteases (11). DPP4 was identified as a functional receptor of Middle East respiratory syndrome coronavirus (MERS-CoV) because the receptor-binding domain (RBD) of the MERS-CoV spike protein specifically copurified with DPP4 from cell lysates. The cleavage site of DPP4 was distant from the MERS-CoV RBD binding site, similar to the structure of ACE2 binding to the SARS-CoV RBD (12). Furthermore, a docked model of SARS-CoV-2 predicted that its spike protein S1 domain interacted with DPP4 residues in close proximity to the active region of the S1 domain in SARS-CoV-2. Additional DPP4 residues (Q286, I287, N338, V341, and R336) were also predicted to interact with the S1 domain of the spike protein of SARS-CoV-2 (13).

The mainstay of clinical treatment of COVID-19 consists of symptomatic management with mechanical ventilation for patients with respiratory failure. Although several antiviral drugs, including chloroquine and the nucleotide analog remdesivir, are being actively tested, none have been specifically approved for COVID-19. In addition to vaccine development and approaches that directly target the virus or block viral entry, treatments that address the immunopathology of the infection have become a major focus (14). A better understanding of the relative effects of receptor binding and protease action of DPP4 will help predict whether specific zoonotic coronaviruses infect humans, and the possibility of adaptation.

Here, we demonstrate a landscape profiling analysis on the expression level of DPP4 in healthy tissues and pan-cancers, and the correlation of DPP4 with the infiltration of immune cells in specific tumor patients, to explore the potential implication of DPP4 in tumor patients in terms of SARS-CoV-2 infection. These findings may provide important vigilance clues for specific cancer patients to prevent SARS-CoV-2 infection and alleviate the cytokine storm in infected patients. Given that DPP4 inhibitors have been used as clinical therapies for diabetes (such as sitagliptin, saxagliptin, and vildagliptin) (15), this study might shed light on the therapeutic potential of DPP4 inhibitors for preventing or attenuating SARS-CoV-2 infection in specific tumor patients.

## Methods

### Tissue Atlas Database

The Tissue Atlas database (https://www.proteinatlas.org/humanproteome/tissue) contains numerous expression profiles of human genes both at the mRNA and protein levels expressed in specific tissues and organs. The expression of tissues-restricted genes is defined as being significantly elevated more than four-fold in specific tissues compared to other tissues. The mRNA expression data was acquired from deep sequencing of RNA (RNA-seq) in 37 different types of normal tissues and organs; and there are 15,313 protein sequences were available in the protein dataset. The expression of proteins was validated in 44 normal tissues using immunohistochemistry, and all immunohistochemistry images were available in the Tissue Atlas database. All data are available online.

### Blood Atlas Analysis

The Blood Atlas database (https://www.proteinatlas.org/humanproteome/blood) contains single-cell type information on RNA expression profiles in 18 immune cell types, including B cells, T cells, monocytes, granulocytes, dendritic cells, and others. DPP4 RNA expression in these blood immune cells, and DPP4 protein detected in human plasma using mass spectrometry-based proteomics were analyzed in this database. All data are available online.

### ENCORI Dataset Analysis

The ENCORI database (http://starbase.sysu.edu.cn/panCancer.php) contained the most comprehensive pan-cancer expression map and interaction network, accompanied by gene expression data of 32 types of cancers, which were derived from 10,882 RNA-seq and 10,546 miRNA-seq data. Differential expression of DPP4 in pan-cancers was analyzed automatically when we entered the gene symbol into the search line. DPP4 expression in clinical cancer specimens was compared with that in paired normal controls using the Student’s t-test. Kaplan–Meier and log-rank methods were used to analyze overall survival. All data are available online.

### UALCAN Database Analysis

The UALCAN database (http://ualcan.path.uab.edu/analysis.html) is a comprehensive and interactive web resource for analyzing cancer OMICS data (16). UALCAN was used to explore the expression of DPP4 in lung adenocarcinoma (LUAD) based on sample types, individual cancer stages, patient age, and nodal metastasis status. The DPP4 promoter methylation level in different types of cancer was also explored in the UALCAN database.

### HCCDB Database Analysis

The HCCDB database (http://lifeome.net/database/hccdb/home.html) is an archive of 15 public hepatocellular carcinoma (HCC) gene expression datasets containing 3,917 samples. For 13 microarray datasets, probe values (log2 intensity) and probe annotations were extracted from raw files downloaded from the Gene Expression Omnibus (GEO) database (17). HCCDB was also used to search for the DPP4 gene in normal tissues and pan-cancers.

### TIMER Database Analysis

TIMER is a comprehensive database for systematic analysis of immune infiltrates across diverse cancer types (https://cistrome.shinyapps.io/timer/) (18), applying a deconvolution statistical method to infer the abundance of tumor-infiltrating immune cells from gene expression profiles. The TIMER database contains 10,897 samples across 32 cancer types from The Cancer Genome Atlas (TCGA) to estimate the infiltration of immune cells. DPP4 and ACE2 expression in lung squamous cell carcinoma (LUSC) and the correlation of DPP4 or ACE2 expression with the abundance of immune infiltrates, including B cells, CD4^+^T cells, CD8^+^T cells, neutrophils, macrophages, and dendritic cells, by gene modules were analyzed in TIMER. Correlations between DPP4 or ACE2 expression and gene markers of tumor-infiltrating immune cells were explored using correlation modules. The correlation module generated expression scatter plots between a pair of user-defined genes in a given cancer type, together with the Spearman’s correlation, and the estimated statistical significance.

### Potential Correlation Between DPP4 and C-X-C Motif Chemokine Ligand 10 (CXCL10) in GENEMANIA

GENEMANIA (http://genemania.org) is an online tool that can predict gene function and identify the genes most closely related to a query gene set using a guilt-by-association approach. The GENEMANIA indexes 2,277 association networks containing 597,392,998 interactions mapped to 163,599 genes from nine organisms. The potential correlation between DPP4 and CXCL10 was explored in GENEMANIA. The correlation was demonstrated from the aspects of physical interactions, co-expression, predicted, co-localization, pathway, genetic interactions, and shared protein domains.

### Expression of DPP4 in Bronchial Epithelial Cells Infected With SARS-CoV-2

To investigate DPP4 expression in SARS-CoV-2 infected versus non-infected epithelial cells, we re-analyzed the publicly available transcriptomic dataset (GSE147507) in Gene Expression Omnibus (GEO) (19). Independent biological triplicates of primary human lung epithelium (NHBE) were mock-treated or infected with SARS-CoV-2 (USA-WA1/2020), then subjected to whole transcriptomic analysis using RNA-Sequencing on Illumina Next Seq 500. Graphical visualization of the DPP4 expression was made using the Graph Prism 7.0.

### Statistical Analysis

The results of ENCORI and UALCAN are displayed with hazard ratio (HR) and P-value or Cox P-values from a log-rank test. The correlation of gene expression was evaluated by Spearman’s correlation and statistical significance, and the strength of the correlation was determined using the following guide for the absolute value: 0.00–0.19 “very weak,” 0.20–0.39 “weak,” 0.40–0.59 “moderate,” 0.60–0.79 “strong,” and 0.80–1.0 “very strong.” P-values <0.05 were considered statistically significant.

## Results

### Expression Profile of DPP4 in Healthy Human Tissues

**Figure 1:**
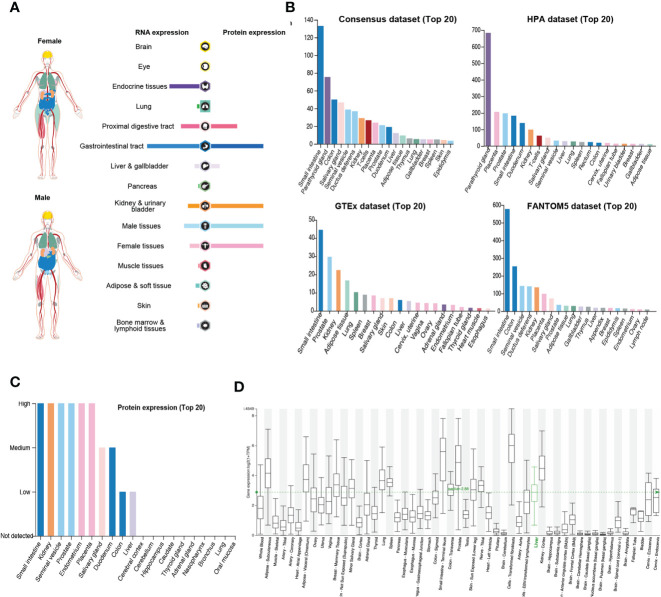
Expression of DPP4 in human tissues and organs. **(A)** mRNA and protein expression profiles of DPP4 in all tissues and organs analyzed in the Tissue Atlas database; **(B)** Top 20 tissues or organs with DPP4 mRNA expression in four databases (Consensus dataset, Fantom5 dataset, GTEx dataset, and HPA dataset); **(C)** TOP20 tissues or organs with DPP4 protein expression in related database. **(D)** DPP4 mRNA expression in various tissues analyzed in the HCCDB database. DPP4 (CD26), dipeptidyl peptidase 4.

DPP4 is a potential receptor of SARS-CoV-2; therefore, we first determined an overview of DPP4 expression in healthy human tissues. DPP4 gene and protein expression were more enriched in the gastrointestinal tract, proximal digestive tract, kidney, and urinary bladder in male and female tissues, with the protein levels being higher than the RNA levels (**Figure 1A**). We further analyzed DPP4 gene expression in four datasets (Consensus, HPA, GTEx, and FANTOM5) and listed the top 20 tissues with high *DPP4* expression (**Figure 1B**). The small intestine had the highest expression of *DPP4* in three of the datasets, and the parathyroid gland was reported to have the highest expression in the HAP dataset. Prostate, colon, and kidney also showed relatively higher expression of *DPP4* compared to other organs. It is intriguing that adipose tissue demonstrated abundant *DPP4* expression in the GTEx dataset, which is consistent with the current concept that obese patients are susceptible to SARS-CoV-2 infection (**Figure 1B**). At the protein level, DPP4 was enriched in the digestive and urinary systems, including the small intestine, kidney, seminal vesicle, prostate, endometrium, placenta, salivary gland, duodenum, colon, and liver (**Figure 1C**). Moreover, by analyzing the HCCDB database, we confirmed DPP4 gene expression in various tissues and found that the small intestine had the highest DPP4 gene expression, which was in-line with the expression of *ACE2* (9), that small intestine showed the most abundant expression of ACE2, and colon and kidney exhibited a relatively higher expression compared to other organs (**Supplementary Figure 1**). It is worth noting that subcutaneous and visceral adipose tissues expressed relatively high levels of DPP4 compared to that of the lung, prostate, and kidney (**Figure 1D**). Taken together, DPP4 is expressed in various healthy human tissues, especially in the digestive and urinary systems as well as in adipose tissue, showing the potential susceptibility to SARS-CoV-2 infection. In order to identify DPP4 expression in SARS-CoV-2 infected *versus* non-infected epithelial cells, we re-analyzed the publicly available transcriptomic dataset (GSE147507) recently uploaded to the GEO (19). The mRNA level of DPP4 in lung of patient infected with SARS-CoV-2 significantly decreased *versus* non-infected healthy control (**Supplementary Figure 2A**). The A549 cell line was further transfected with ACE2 promoter and then exposed to mock, SARS-CoV-2 or SARS-CoV-2 plus Ruxolitinib (ACE2 inhibitor). Gene expression of DPP4 was dramatically decreased in A549 cells infected with SARS-CoV-2 in the presence or absence of Ruxolitinib (**Supplementary Figure 2B**). The tendency of DPP4 was similar to that of ACE2 in epithelial cells with SARS-Cov-2 infection (8). In summary, these data together with previous findings strongly suggested that DPP4 has the great potential in mediating the infection of SARA-Cov-2.

### Expression and Prognostic Potential of DPP4 in Pan-Cancers

**Figure 2:**
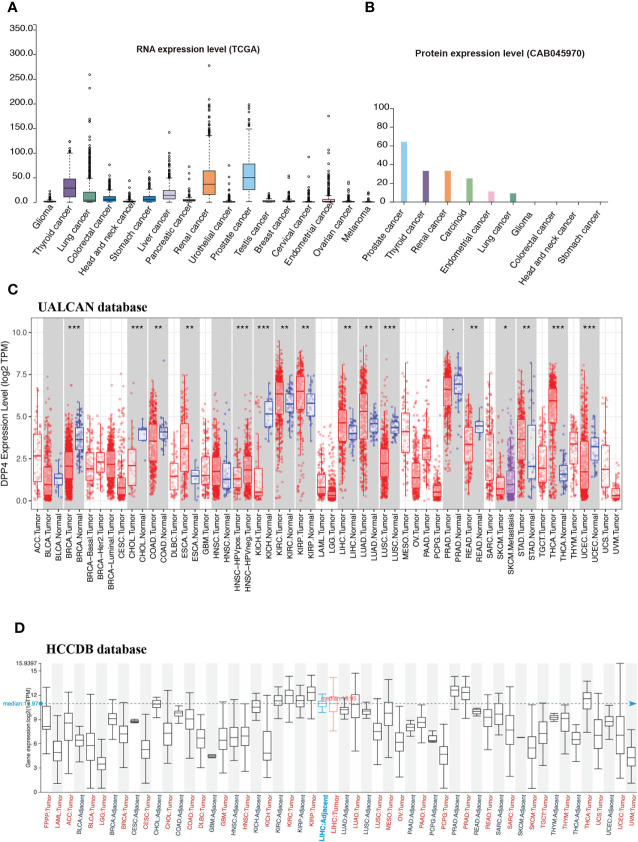
Expression profile of DPP4 in pan-cancers. **(A)** RNA expression level of DPP4 in pan-cancers in TCGA dataset. **(B)** Protein expression level of DPP4 in pan-cancer in CAB045970. **(C)** RNA expression level of DPP4 in pan-cancers analyzed in UALCAN database. **(D)** RNA expression level of DPP4 in pan-cancers analyzed in HCCDB database. DPP4, dipeptidyl peptidase 4.

Cancer patients are more susceptible to developing novel coronavirus pneumonia and are at risk of poor prognosis after infection with SARS-CoV-2 due to impaired immunity (14). Thus, we next estimated the expression profiles of the potential SARS-CoV-2 receptor DPP4 in pan-cancers. In the TCGA dataset, we found that *DPP4* was highly expressed in renal cancer and prostate cancer, which was consistent with its expression in normal tissues (**Figure 2A**). In addition, the DPP4 protein expression level was slightly different from that of RNA. Prostate cancer was associated with the most abundant expression of *DPP4*, followed by thyroid cancer, renal cancer, carcinoid, endometrial cancer, and lung cancer; DPP4 was ordinarily analyzed in CAB045970 database (**Figure 2B**). To further evaluate DPP4 expression in human cancers, we examined RNA-seq data of multiple malignancies in TCGA analyzed by UALCAN (**Figure 2C**), HCCDB (**Figure 2D**), and ENCORI (**Table 1**). DPP4 expression was significantly lower in breast invasive carcinoma, kidney chromophobe, cholangiocarcinoma, colon adenocarcinoma, and LUSC. However, increased DPP4 expression was observed in LUAD, thyroid carcinoma, and uterine corpus endometrial carcinoma compared with adjacent normal tissues (P < 0.05; false discovery rate [FDR] < 0.05). LUAD has been reported as the most frequent cancer type associated with SARS-CoV-2 infection among malignancies (4), and it was confirmed to harbor a higher incidence of COVID-19 with more severe symptoms (4). Here, we showed that *DPP4* expression was upregulated and positively associated with age, cancer stage, and nodal metastasis in patients with LUAD (**Supplementary Figure 3**). These results suggest that age and/or advanced cancer increase the vulnerability of these patients to SARS-CoV-2 infection.

**Table 1:** Expression of DPP4 in different types of cancer compared with normal samples.

Cancer	Cancer full name	Cancer number	Normal number	Cancer Exp	Normal Exp	Fold change	P value	FDR
BLCA	Bladder Urothelial Carcinoma	411	19	2.12	0.99	2.14	0.49	0.74
BRCA	Breast Invasive Carcinoma	1104	113	2.3	6.34	0.36	9.00E-36	1.50E-34
CHOL	Cholangiocarcinoma	36	9	5.17	9.09	0.57	0.0022	0.0074
COAD	Colon Adenocarcinoma	471	41	9.16	11.6	0.79	0.014	0.039
ESCA	Esophageal Carcinoma	162	11	7.25	5.1	1.42	0.096	0.3
HNSC	Head and Neck Squamous Cell Carcinoma	502	44	2.09	3.77	0.56	0.89	0.92
KICH	Kidney Chromophobe	65	24	4.93	18.27	0.27	1.50E-11	1.60E-10
KIRC	Kidney Renal Clear Cell Carcinoma	535	72	42.85	27.22	1.57	0.17	0.23
KIRP	Kidney Renal Papillary Cell Carcinoma	289	32	56.5	29.76	1.9	0.22	0.41
LIHC	Liver Hepatocellular Carcinoma	374	50	18.95	9.68	1.96	0.056	0.12
LUAD	Lung Adenocarcinoma	526	59	29.39	11.06	2.66	0.051	0.1
LUSC	Lung Squamous Cell Carcinoma	501	49	3.77	9.68	0.39	3.30E-22	3.70E-21
PAAD	Pancreatic Adenocarcinoma	178	4	5.59	2.78	2.01	0.47	0.94
PRAD	Prostate Adenocarcinoma	499	52	55.2	61.96	0.89	0.13	0.27
STAD	Stomach Adenocarcinoma	375	32	9.15	14.82	0.62	0.046	0.086
THCA	Thyroid Carcinoma	510	58	31.4	1.45	21.63	3.40E-36	4.10E-34
UCEC	Uterine Corpus Endometrial Carcinoma	548	35	7.57	4.93	1.54	0.0067	0.022

DPP4, Dipeptidyl peptidase 4; Exp, Expression level; FDR, false discovery rate.

**Figure 3:**
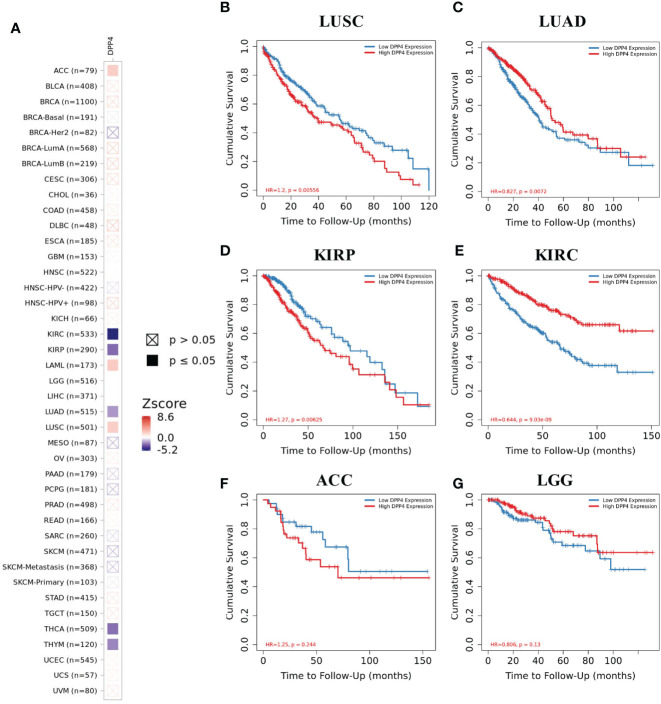
Efficiency of DPP4 expression in the survival of pan-cancer patients. **(A)** Co-efficiency of DPP4 expression with the survival of pan-cancer patients analyzed with TIMER2. **(B−G)** Kaplan-Meier Plotter of LUSC, LUAD, KIRP, KIRC, ACC, and LGG based on the expression level of DPP4. DPP4, dipeptidyl peptidase 4; LUSC, lung squamous cell carcinoma; LUAD, lung adenocarcinoma; KIRP, kidney renal papillary cell carcinoma; KIRC, kidney renal clear cell carcinoma; ACC, adrenocortical carcinoma; LGG, low grade glioma. Spearman’s ρ (red): positive correlation, p < 0.05, *ρ* > 0; Spearman’s *ρ* (blue): negative correlation, p < 0.05, *ρ* < 0.

We next examined the efficiency of DPP4 in the survival of pan-cancer patients with TIMER. We found that decreased DPP4 expression was significantly associated with poor prognosis in overall survival of patients with kidney renal clear cell carcinoma (KIRC; z_score = −5.162245798), LUAD (z_score = −2.004922368), thyroid carcinoma (z_score = −2.837080854), and thymoma (z_score = −2.373081403). On the other hand, elevated DPP4 expression correlated with poor prognosis in adrenocortical carcinoma (z_score = 2.047142556), acute myeloid leukemia (LAML; z_score = 2.234472636), brain lower grade glioma (LGG,z_score = 8.609548747), kidney renal papillary cell carcinoma (KIRP; z_score = −2.987857684), and LUSC (z_score = 2.103042397) (**Figure 3A**, **Supplementary Table 1**). The Kaplan–Meier Plotter of LUSC, LUAD, KIRP, KIRC, brain lower grade glioma, and adrenocortical carcinoma further showed that DPP4 expression significantly correlated with the prognosis of specific cancer types (**Figures 3B–G**). The distinct role of DPP4 in these specific cancers might be due to the particular distribution of DPP4 in different cell types.

The DNA methylation level of DPP4 was further analyzed in several types of cancer. We found that four tumor types with the highest DPP4 expression presented with a decreased level of DPP4 DNA methylation, including prostate adenocarcinoma, thymoma, bladder cancer, and KIRC. Accordingly, in a DPP4 downregulated tumor such as LUSC, increased DNA methylation levels were observed. However, in KIRP, increased DPP4 expression was accompanied by elevated DNA methylation of DPP4 (**Supplementary Figure 4**), suggesting that DNA methylation might not be the only reason for abnormal DPP4 expression. Since our study is only a database analysis, validation in a larger clinical cohort is warranted.

### DPP4 Is Expressed Abundantly in Both Innate and Adaptive Immune Cells

**Figure 4:**
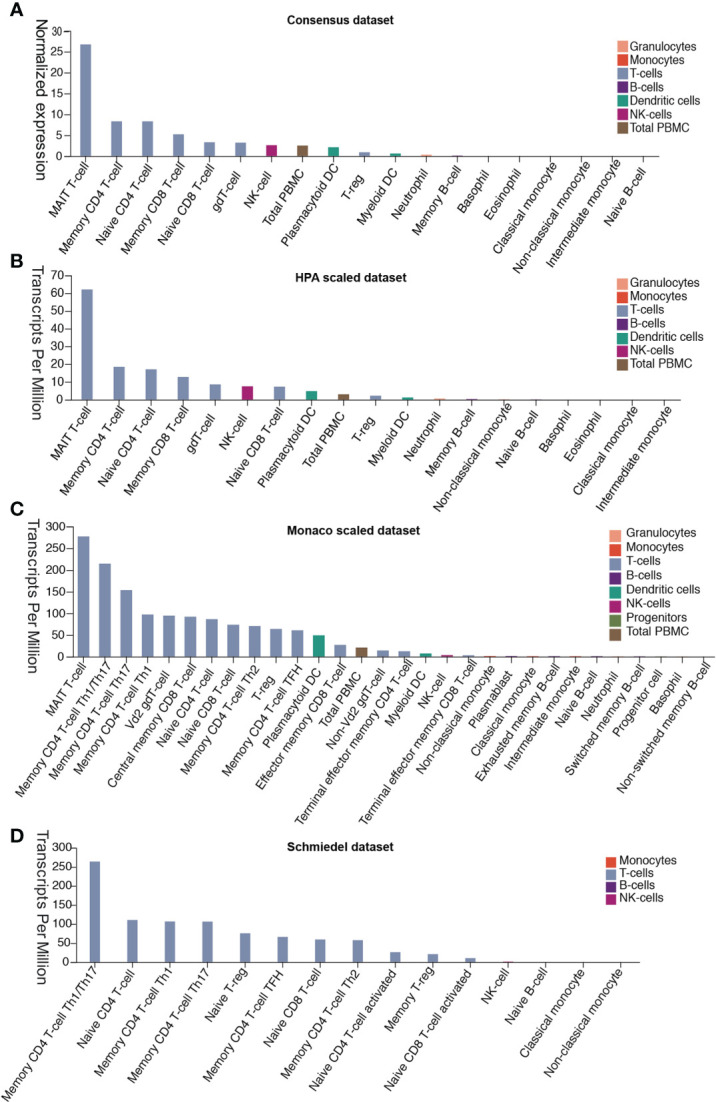
DPP4 expression in human blood cells analyzed in four datasets. **(A)** Consensus dataset, **(B)** HPA scaled dataset, **(C)** Monaco scaled dataset, and **(D)** Schmiedel dataset. DPP4, dipeptidyl peptidase 4.

Patients with severe SARS-CoV-2 infection often experience a cytokine storm (6, 20). The cytokine storm can initiate viral sepsis and inflammatory-induced lung injury, which lead to other complications including pneumonitis, ARDS, respiratory failure, shock, organ failure, and potentially death (21). We first investigated whether the release of these abnormal cytokines was associated with the expression of DPP4 in various immune cells in blood from four different datasets. Analysis of the data demonstrated that mucosal-associated invariant T cells, the unique innate-like T cells that bridge innate and adaptive immunity, exhibited the highest expression of DDP4, which might be responsible for fecal-oral transmission of SARS-CoV-2. Memory CD4^+^ T cells, memory CD8^+^ T cells, native CD4^+^ T cells, native CD8^+^ T cells, gamma delta T cells, and regulatory T (Treg) cells also expressed appreciable levels of DPP4. Moreover, DPP4 was also expressed in other immune cells, including natural killer (NK) cells, peripheral blood mononuclear cells, plasmacytoid dendritic cells, myeloid dendritic cells, neutrophils, and memory B cells, as indicated by the Consensus dataset analysis, including the HPA scaled dataset, Monaco scaled dataset, and Schmiedel dataset (**Figures 4A–D**). These data demonstrated that DPP4 is expressed in various innate and adaptive immune cells, especially in T cells, suggesting that DPP4 plays an integral role in the immune system, particularly in T cell activation, which may account for the cytokine storm in SARS-CoV-2-infected patients.

### DPP4 Expression Is Correlated With Immune Cell Infiltration in Pan-Cancer Patients

**Figure 5:**
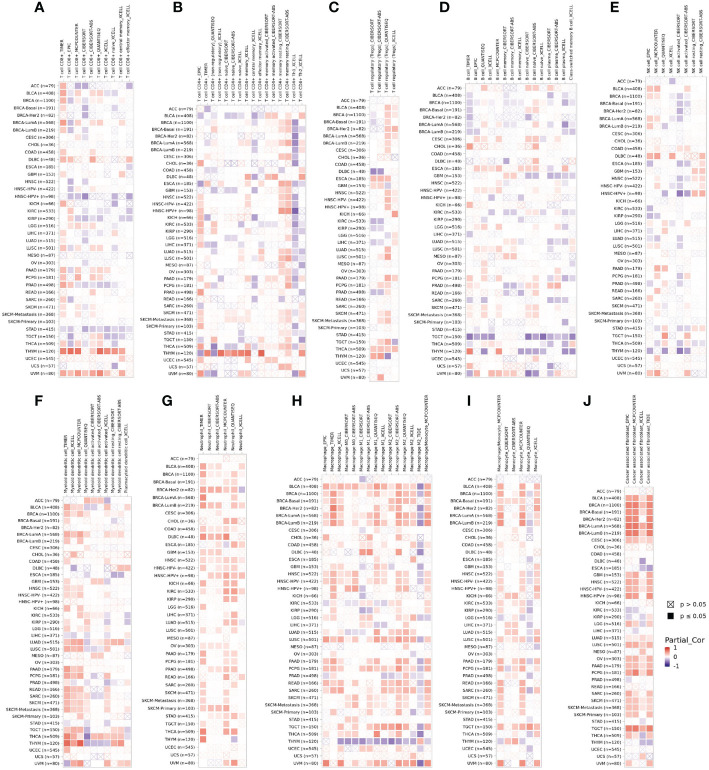
DPP4 correlates with the infiltration of innate and adaptive immune cells in various cancer types. Correlation of DPP4 with **(A)** the infiltration of CD8^+^ T cells, **(B)** CD4^+^ T cells, **(C)** Treg cells, **(D)** B cells, **(E)** NK cells, **(F)** dendritic cells, **(G)** neutrophils, **(H)** macrophages, **(I)** monocytes, and **(J)** cancer associated fibroblasts in various cancer types analyzed by TIMER2 after purity adjustment. Spearman’s *ρ* (red): positive correlation, p < 0.05, *ρ*>0; Spearman’s *ρ* (blue): negative correlation, p < 0.05, *ρ* <0.

Tumor-infiltrating immune cells are independent predictors of sentinel lymph node status and survival in cancers (22, 23). Therefore, we investigated whether DPP4 expression was correlated with immune infiltration levels in various cancer types from TIMER. The results showed that DPP4 expression significantly correlated with infiltration of CD8^+^ T cells (**Figure 5A**), CD4^+^ T cells (**Figure 5B**), Treg (**Figure 5C**), B cells (**Figure 5D**), NK cells (**Figure 5E**), dendritic cells (**Figure 5F**), neutrophils (**Figure 5G**), macrophages (**Figure 5H**), monocytes (**Figure 5I**), and cancer-associated fibroblasts (**Figure 5J**) in pan-cancer patients.

**Figure 6:**
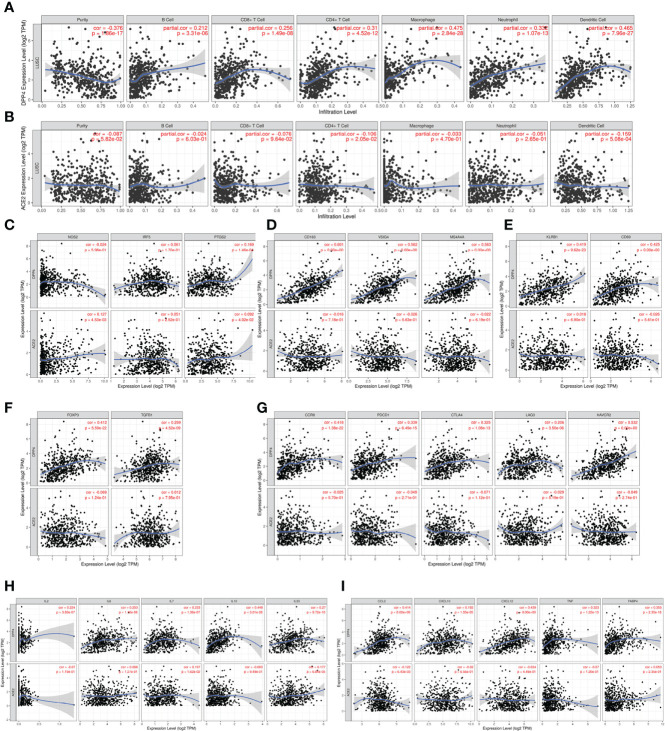
DPP4 promotes the infiltration of immune cells and the release of cytokines in lung squamous cell carcinoma (LUSC) analyzed with TIMER. **(A–B)** Correlation of **(A)** DPP4 and **(B)** ACE2 expression with the infiltration levels of different immune cells (B, CD8+T, CD4+T, macrophages, neutrophils, and dendritic cells) in LUSC analyzed with TIMER. **(C–F)** Scatterplots of correlations between DPP4 and ACE2 expression with gene markers of **(C)** M1 macrophages, **(D)** M2 macrophage, **(E)** memory T cells, and **(F)** regulatory T cells in LUSC. **(G)** Scatterplots of correlations between DPP4 expression, ACE2 expression and T cell exhaustion markers, respectively. **(H)** Correlation of DPP4 and ACE2 with the expression of interleukins (IL2, IL6, IL7, IL10, and IL33) in LUSC. **(I)** Correlation of DPP4 and ACE2 with the expression of cytokines (CCL2, CXCL10, CXCL12, TNF and FABP4) in LUSC, respectively. DPP4 or ACE2 was used for the y-axis with gene symbols and on the x-axis, related marker genes are represented as gene symbols. The gene expression level was displayed using log2 RSEM. DPP4, dipeptidyl peptidase 4; ACE2, angiotensinI-converting enzyme 2.

Since SARS-Cov-2 mainly causes ‘immune storm’ and pneumonia in patients, and the expression of DPP4, an alternative receptor of SARS-Cov-2, is a prognostic factor for LUSC patients. High expression of DPP4 may associate with elevated morbidity of COVID-19 and poor prognosis in LUSC patients, indicating that intensive care is needed for these patients during COVID-19 epidemic. Therefore, we explored whether DPP4 expression correlated with an immune storm in tumor patients such as LUSC. DPP4 expression showed significant correlations with tumor purity in LUSC and significant correlations with the infiltration of B cells, CD8^+^ T cells, CD4^+^ T cells, macrophages, neutrophils, and dendritic cell. DPP4 expression levels were significantly and positively correlated with infiltrating levels of B cells (r = 0.212, P = 3.31e-06), CD8^+^ T cells (r = 0.256, P = 1.49e-08), CD4^+^ T cells (r = 0.31, P = 4.52e-12), macrophages (r = 0.475, P = 2.84e-28), neutrophils (r = 0.332, P = 1.07e-13), and dendritic cells (r = 0.465, P =7.96e-27) in LUSC (**Figure 6A**). These findings strongly suggest that DPP4 may play a specific role in immune infiltration in LUSC, especially in macrophages and dendritic cells.

**Figure 7:**
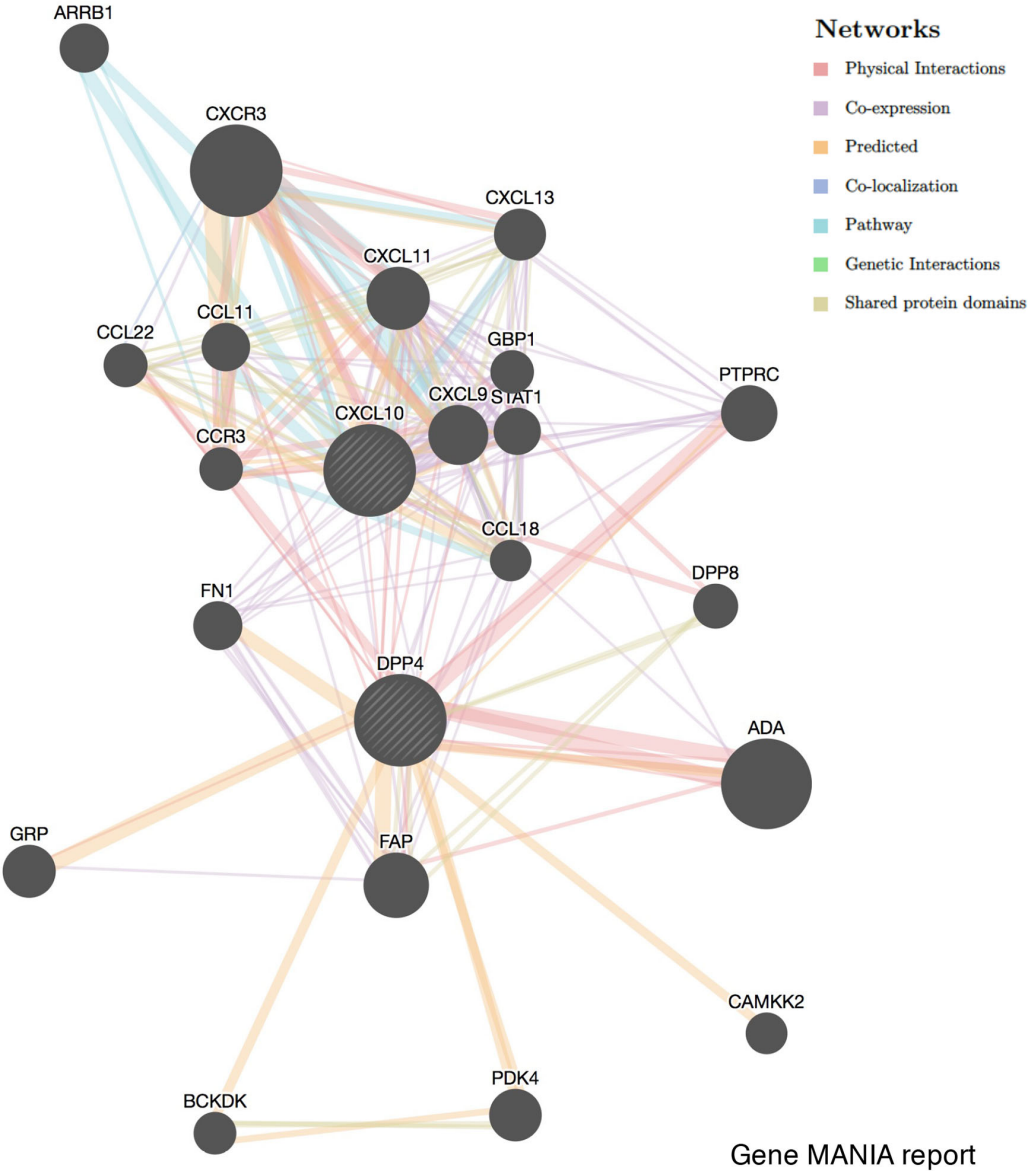
Correlation network of DPP4 and CXCL10 analyzed by the GENEMANIA database. Correlations are demonstrated with different colored lines.

To investigate the relationship between DPP4 and the diverse immune infiltrating cells, we focused on the correlations between DPP4 and immune marker sets of various immune cells of LUSC in the TIMER databases. Interestingly, we found that the expression levels of most marker sets of tumor-associated macrophages, M2 macrophages, but not M1 macrophages, were strongly correlated with DPP4 expression in LUSC. Specifically, we showed that CD163 (r = 0.601, P = 0e+00), VSIG4 (r = 0.562, P = 0e+00), and MS4A4A (r = 0.563, P = 0e+00) of the M2 phenotype were significantly correlated with DPP4 expression in LUSC (**Figures 6B, C** **)**. Since DPP4 was abundantly expressed in memory T cells, we explored the correlation of DPP4 with the infiltration of memory T cells classified with KLRB1 (r = 0.419, P = 9.62e-23) and CD69 (r = 0.425, P = 0.00e+00) (**Figure 6D**). Moreover, DPP4 was also positively correlated with the infiltration of Treg cells with FOXP3 (r = 0.412, P = 5.59e-22) and TGFB1 (r = 0.259, P = 4.52e-09) (**Figure 6E**). We also found significant correlations between DPP4 and marker genes of T cell exhaustion, such as CCR8 (r = 0.418, P = 1.38e-22), PD-1(r = 0.339, P = 6.49e-15), CTLA4(r = 0.325, P = 1.08e-13), LAG3(r = 0.206, P = 3.50e-06), and HAVCR2(r = 0.532, P = 0.00e+00) (**Figure 6F**). Interestingly, HAVCR2 is a crucial gene that regulates T cell exhaustion and exhibited a moderately positive correlation with DPP4 expression, suggesting that high DPP4 expression may play an important role in HAVCR2 mediating T cell exhaustion. These results further supported that DPP4 was significantly correlated with immune infiltrating cells in LUSC, which might contribute to the immune escape in LUSC. We further showed that DPP4 expression significantly correlated with the levels of several critical proinflammatory cytokines and chemokines, including IL-2 (r = 0.224, P = 3.83e-07), IL-6 (r = 0.253, P = 1.10e-08), IL-7 (r = 0.233, P = 1.36e-07), IL-10 (r = 0.449, P = 3.01e-26), IL-33 (r = 0.27, P = 9.72e-10), CCL2 (r = 0.414, P = 0.00e+00), CXCL10 (r = 0.192, P = 1.55e-05), CXCL12 (r = 0.439, P = 0.00e+00), TNFα (r = 0.323, P = 1.22e-13), and FABP4 (r = 0.355, P = 2.35e-16) (**Figures 6G, H** **)**. As ACE2 is the identified receptor for SARS-Cov-2, we also explored the correlation of ACE2 with immune cells infiltration in LUSC together with DPP4. It was interesting to find that ACE2 showed quite weak correlation with immune infiltration comparing to that of DPP4 (**Figure 6**), moreover, the correlation of DPP4 with ACE2 in LUSC was also weak and had no significance (cor = −0.005, P=9.09e-1) (**Supplementary Figure 5**), suggesting that DPP4 but not ACE2, might be responsible for the outbreak of cytokine storm in LUSC patients. Furthermore, CXCL10 has shown great anti-tumor potential (24); thus, we further showed that DPP4 was correlated with CXCL10, a novel anti-tumor chemokine, through physical interaction and co-expression by GENEMANIA analysis (**Figure 7**). Taken together, these data suggested that expression of DPP4 may be closely associated with immune infiltration in specific cancers patients, which might also contribute to the cytokine storm in SARS-CoV-2 infected patients.

## Discussion

COVID-19 is now a worldwide pandemic; however, there are currently no effective therapeutic strategies. What is even worse is that obese individuals and cancer patients are both more vulnerable to SARS-CoV-2 infection and a poor prognosis due to the high expression of ACE2, the SARS-CoV-2 receptor (8, 9, 25). However, apart from the intensive studies on ACE2, understanding additional potential receptors of SARS-CoV-2 may also be helpful in offering novel insights and potential therapeutic targets for combating SARS-CoV-2 infection.

DPP4 is a functional receptor of MERS-CoV; the receptor-binding S1 domain of the MERS-CoV spike protein was copurified with DPP4 specifically from lysates of susceptible Huh-7 cells. The cleavage site of DPP4 was distant from the MERS-CoV RBD site, similar to the structure of ACE2 binding to the SARS-CoV RBD (12). Furthermore, Vankadari et al. predicted the glycan shield and structure prediction of the spike glycoprotein in SARS-CoV-2 and its interaction with DPP4 (13). Expression of DPP4 declined in the lung tissue of SARS-CoV-2 infected patients compared to that of normal healthy controls (19). In addition, the distribution of DPP4 in healthy individuals was similar to that of ACE2 (**Figure 1**). We found that DPP4 was most abundantly expressed in the digestive system, especially in the small intestine and colon, which might contribute to the fecal-oral route transmission of SARS-CoV-2. It is worth noting that the majority of patients infected with SARS-CoV-2 also had renal dysfunction, which might be explained by the high expression of DPP4 in the kidney and urinary system (26, 27). Furthermore, DPP4 levels increased significantly in circulation and adipose tissue in obese patients (22, 23) and some specific tumor patients (**Figure 2**, **Table 1**). LUAD has been reported as the most frequent cancer type in patients infected with COVID in terms of malignancies (4), and elevated DPP4 was detected consistently in LUAD patients and correlated with patient age, individual cancer stage, and nodal metastasis status (**Supplementary Figure 2**). It is worth to note that DPP4 expression increased at early stage of LUAD patients while decreased at late stage. As DPP4 is closely associated with tumor immunity (28), it might because of the diminished immune response at terminal stage of tumor patients. Although the expression of DPP4 doesn’t demonstrate the significant upregulation in comparison to other types of cancer, it might contribute to the high infection rate in LUAD patients apart from the abundant expression of ACE2 (29). These findings suggest that DPP4 might be one of the most promising receptors of SARS-CoV-2 beyond ACE2.

We further showed the risk of SARS-CoV-2 infection according to different types of cancers and the expression level of DPP4. High DPP4 expression indicated a poor prognosis in some specific cancer types, including LUSC, bladder cancer, prostate adenocarcinoma, and brain lower grade glioma, and we found that the unique cancer LUSC was associated with DPP4 expression and prognosis (**Figure 3**, **Supplementary Table 1**). Our study provided potential clues for preventing SARS-CoV-2 infection in patients with cancer. For example, utilization of a DPP4 inhibitor might be a promising strategy in LUSC patients; it would not only reduce the susceptibility to SARS-CoV-2 infection, but also increase the survival rate in LUSC patients.

Based on previous clinical data, the majority of COVID-19 cases (about 80%) presented with asymptomatic or mild symptoms, while the remainder were patients with severe or critical cases (2, 30). Furthermore, high levels of proinflammatory cytokines were observed in COVID-19 severe cases (2). This cytokine storm can initiate viral sepsis and inflammatory-induced lung injury, which can lead to other complications including pneumonitis, ARDS, organ failure, and potentially death, indicating that the cytokine storm may have a major role in the pathogenesis of COVID-19 (6, 20). In this study, we demonstrated that DPP4 is expressed in many types of immune cells, including T cells, NK cells, and dendritic cells. DPP4 was especially abundant in mucosal-associated invariant T cells, memory CD4 T cells, and its subtypes Th1, Th2, Th17, and Treg cells. High expression of DPP4 was also detected in native and memory CD8^+^ T cells analyze in four datasets (**Figure 4**). The expression differences of DPP4 in various immune cells between the datasets might be attributed to the effects of algorithms on obtaining different expression levels in different cell types.

Previous studies have shown that virus-specific T cells from the severe group tended to be a central memory phenotype with a significantly higher frequency of polyfunctional CD4^+^ T cells and CD8^+^ T cells after SARS-CoV infection (21). In MERS-CoV infection, the early rise of CD8^+^ T cells correlates with disease severity and at the convalescent phase, are accompanied by dominant Th1 type helper T cells (31). Airway memory CD4^+^ T cells specific for the conserved epitope are protective against lethal challenge and can cross-react with SARS-CoV and MERS-CoV in animal models (32). Using the TIMER analyzer, we found that DPP4 expression was correlated with diverse immune infiltration levels in various cancer types (**Figure 5**). Moreover, the correlation between DPP4 expression and the marker genes of immune cells implicates the role of DPP4 in regulating tumor immunology in LUSC (**Figure 6**). Gene markers of M1 macrophages showed weak correlations, whereas M2 macrophage markers showed moderate and strong correlations with DPP4 expression, revealing the potential regulatory role of DPP4 in the polarization of tumor-associated macrophages. Moreover, DPP4 showed the potential to activate memory T cells (T_mem_) and Tregs and to induce the exhaustion of T cells. In view of the profiles of DPP4 expression and the correlation with immune cells and various pro-inflammatory cytokines and chemokines in LUSC patients (**Figure 6**), DPP4 might have a great potency in exacerbating the cytokine storm in specific cancer patients after SARS-CoV-2 infection.

DPP4 is also a key immunoregulatory factor for hijacking and virulence. It has the capacity to selectively degrade various substrates, including incretin hormones, growth factors, and cytokines, by cleaving dipeptides from the amino N-terminus. Apart from enzymatic activity, DPP4 is also involved in a variety of biological processes, including regulation of the immune system, including T cell activation, chemotaxis regulation, cell adhesion, apoptosis, and tumorigenicity (11, 28). DPP4 can work on many types of cells, including T lymphocytes, adipocytes, hepatocytes, and smooth muscle cells, which triggers metabolic and immune responses in cancer patients. Elevated circulatory DPP4 levels and activity have been found in a wide spectrum of metabolic syndromes, including diabetes, obesity, cardiovascular diseases, and nonalcoholic fatty liver diseases. DPP4 inhibitors such as sitagliptin, saxagliptin, and vildagliptin are now in clinical use as antidiabetic drugs and act by prolonging the insulinotropic effect of incretins through glucagonlike peptide-1 and the glucose-dependent insulinotropic polypeptide signaling pathway (33). Recent preclinical studies have further expanded the repertoire for the use of DPP4 inhibitors in the treatment of other metabolic diseases and their consequent complications (34), which may block the signaling pathway of DPP4 with NFκB and matrix metallopeptidase 9 in these patients (11, 34). In patients with tumors, apart from activating T cell maturation and priming, post-translational modification of chemokines mediated by DPP4 negatively regulates lymphocyte trafficking, and its inhibition enhances T cell migration and tumor immunity by preserving the functional chemokine CXCL10 (35). Furthermore, inhibition of DPP4 reveals IL-33-dependent eosinophil-mediated control of tumor growth. Administration of the DPP4 inhibitor resulted in higher concentrations of CCL11 and increased migration of eosinophils into tumor parenchyma, enhancing the eosinophil-mediated anti-tumor responses (36). As the expression of DPP4 increased in the adipose tissue of obese patients (22, 23) and some specific cancer patients (**Figure 2**, **Table 1**), it was anticipated to reduce the infection rate of SARS-CoV-2 in patients by treatment with DPP4 inhibitors, while further clinical trials are needed to verify our hypothesis. Solerte et al. found that DPP4 inhibitors, such as gliptins may offer a simple way to reduce the virus entry and replication into the airways and to hamper the sustained cytokine storm and inflammation within the lung in patients diagnosed with COVID-19 infection (37). However the effects of DPP4 inhibitor in COVID-19 infection are still contradictory at present; Dalan et al. claimed that DPP4 inhibitor may not be beneficial in hypertension or diabetes patients infected with COVID-19 (38). Real world evidence and reports of observational evidence of impact of DPP4 inhibitors in tumor patients in the setting of COVID-19 infection are required to make definite conclusions with regards to the whether they are beneficial.

**Figure 8:**
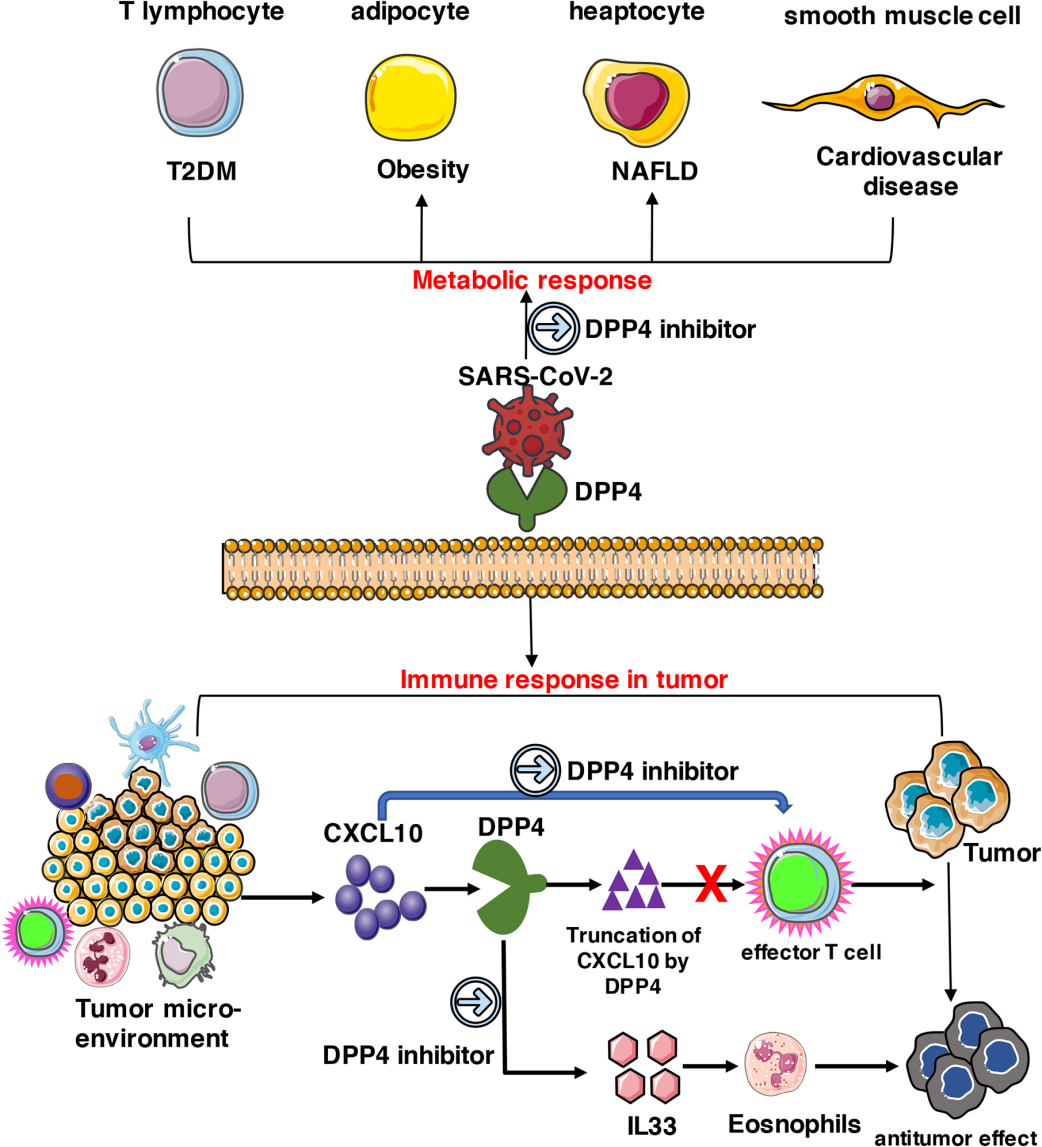
Potential implication of DPP4 inhibitor in preventing SARS-CoV-2 damage in tumor patients. DPP4 expressed abundantly in various types of cells including T lymphocytes, adipocytes, hepatocytes, and smooth muscle cells, which triggers metabolic and immune responses in cancer patients. As a potential receptor for SARS-CoV-2, DPP4 inhibitors might also possess beneficial therapeutic effects on SARS-CoV-2 treatment and work together with other effective strategies.

In summary, DPP4 is abundantly expressed in various types of cells including T lymphocytes, adipocytes, hepatocytes, and smooth muscle cells, which triggers metabolic and immune responses in obesity-related metabolic complications and cancer patients. As a potential receptor for SARS-CoV-2, DPP4 inhibitors might exhibit beneficial therapeutic effects on SARS-CoV-2 treatment together with other effective strategies (**Figure 8**). Animal experiments and clinical trials should be conducted in the future to evaluate whether DPP4 inhibitors have therapeutic potential in preventing or alleviating SARS-CoV-2 infection in obese or specific cancer patients.

## Data Availability Statement

The original contributions presented in the study are included in the article/**Supplementary Material**. Further inquiries can be directed to the corresponding author.

## Author Contributions

LS designed the experiments. YLiu, JL, YLi, XW, and HH conducted the experiments. LS, YLiu, and JL prepared the figures and drafted the manuscript. LS edited the manuscript. All authors contributed to the article and approved the submitted version.

## Funding

This work was supported by grants from the National Natural Science Foundation of China (81700749) and the Sun Yat-sen University Hundred Talents Program (PT19200101).

## Conflict of Interest

The authors declare that the research was conducted in the absence of any commercial or financial relationships that could be construed as a potential conflict of interest.

## Abbreviations

ACE2, angiotensin converting enzyme II; ARDS, acute respiratory distress syndrome; COVID-19, coronavirus disease 2019; CXCL10, C-X-C motif chemokine ligand 10; DPP4, dipeptidyl peptidase 4; KIRC, kidney renal clear cell carcinoma; KIRP, kidney renal papillary cell carcinoma; LUAD, lung adenocarcinoma; LUSC, lung squamous cell carcinoma; MERS-CoV, Middle East respiratory syndrome coronavirus; RBD, receptor-binding domain; SARS-CoV-2, severe acute respiratory syndrome coronavirus 2; TCGA, The Cancer Genome Atlas; TIMER, Tumor Immune Estimation Resource.

## References

[1] World Health Organization Coronavirus disease 2019 (COVID-19): situation report, Vol. 121 (2020). Available at:https://apps.who.int/iris/handle/10665/332156

[2] Huang C Wang Y Li X Ren L Zhao J Hu Y Clinical features of patients infected with 2019 novel coronavirus in Wuhan, China. Lancet (2020) 395:497–506. 10.1016/S0140-6736(20)30183-5 31986264 PMC7159299

[3] Xu Z Shi L Wang Y Zhang J Huang L Zhang C Pathological findings of COVID-19 associated with acute respiratory distress syndrome. Lancet Respir Med (2020) 8:420–2. 10.1016/S2213-2600(20)30076-X PMC7164771 32085846

[4] Liang W Guan W Chen R Wang W Li J Xu K Cancer patients in SARS-CoV-2 infection: a nationwide analysis in China. Lancet Oncol (2020) 21:335–7. 10.1016/S1470-2045(20)30096-6 PMC7159000 32066541

[5] Nicholls JM Poon LL Lee KC Ng WF Lai ST Leung CY Lung pathology of fatal severe acute respiratory syndrome. Lancet (2003) 361:1773–8. 10.1016/S0140-6736(03)13413-7 PMC7112492 12781536

[6] Mahallawi WH Khabour OF Zhang Q Makhdoum HM Suliman BA MERS-CoV infection in humans is associated with a pro-inflammatory Th1 and Th17 cytokine profile. Cytokine (2018) 104:8–13. 10.1016/j.cyto.2018.01.025 29414327 PMC7129230

[7] Zhou P Yang X-L Wang X-G Hu B Zhang L Zhang W A pneumonia outbreak associated with a new coronavirus of probable bat origin. nature (2020) 579:270–3. 10.1038s41586-020-2951-z 10.1038/s41586-020-2012-7 PMC7095418 32015507

[8] Al Heialy S Hachim MY Senok A Abou Tayoun A Hamoudi R Alsheikh-Ali A Regulation of angiotensin converting enzyme 2 (ACE2) in obesity: implications for COVID-19. bioRxiv (2020). 10.1101/2020.04.17.046938 PMC7531362 33071815

[9] Dai Y-J Hu F Li H Huang H-Y Wang D-W Liang Y A profiling analysis on the receptor ACE2 expression reveals the potential risk of different type of cancers vulnerable to SARS-CoV-2 infection. Ann Trans Med (2020) 8(7):481. 10.21037/atm.2020.03.61 PMC7210193 32395525

[10] Wang N Shi X Jiang L Zhang S Wang D Tong P Structure of MERS-CoV spike receptor-binding domain complexed with human receptor DPP4. Cell Res (2013) 23:986. 1016/j.cell.2020.04.026 23835475 10.1038/cr.2013.92 PMC3731569

[11] Klemann C Wagner L Stephan M von Hörsten S Cut to the chase: a review of CD26/dipeptidyl peptidase-4’s (DPP4) entanglement in the immune system. Clin Exp Immunol (2016) 185:1–21. 10.1111/cei.12781 26919392 PMC4908298

[12] Lu G Hu Y Wang Q Qi J Gao F Li Y Molecular basis of binding between novel human coronavirus MERS-CoV and its receptor CD26. Nature (2013) 500:227–31. 10.1038/nature12328 PMC7095341 23831647

[13] Vankadari N Wilce JA Emerging COVID-19 coronavirus: glycan shield and structure prediction of spike glycoprotein and its interaction with human CD26. Emerg Microbes Infect (2020) 9:601–4. 10.1080/22221751.2020.1739565 PMC7103712 32178593

[14] Li X Geng M Peng Y Meng L Lu S Molecular immune pathogenesis and diagnosis of COVID-19. J Pharm Anal (2020) 10(2):102–8. 10.1016/j.jpha.2020.03.001 PMC7104082 32282863

[15] Duez H Cariou B Staels B DPP-4 inhibitors in the treatment of type 2 diabetes. Biochem Pharmacol (2012) 83:823–32. 10.1016/j.bcp.2011.11.028 22172989

[16] Chandrashekar DS Bashel B Balasubramanya SAH Creighton CJ Ponce-Rodriguez I Chakravarthi BV UALCAN: a portal for facilitating tumor subgroup gene expression and survival analyses. Neoplasia (2017) 19:649–58. 10.1016/j.neo.2017.05.002 PMC5516091 28732212

[17] Lian Q Wang S Zhang G Wang D Luo G Tang J HCCDB: a database of hepatocellular carcinoma expression atlas. Genomics Proteomics Bioinformatics (2018) 16:269–75. 10.1016/j.gpb.2018.07.003 PMC6205074 30266410

[18] Li T Fan J Wang B Traugh N Chen Q Liu JS TIMER: a web server for comprehensive analysis of tumor-infiltrating immune cells. Cancer Res (2017) 77:e108–10. 10.1158/0008-5472.CAN-17-0307 PMC6042652 29092952

[19] Blanco-Melo D Nilsson-Payant B Liu W-C Møller R Panis M Sachs D Imbalanced host response to SARS-CoV-2 drives development of COVID-19. Cell (2020) 181(5):1036–45 e9. 10.1016/j.cell.2020.04.026 PMC7227586 32416070

[20] Wong C Lam C Wu A Ip W Lee N Chan I Plasma inflammatory cytokines and chemokines in severe acute respiratory syndrome. Clin Exp Immunol (2004) 136:95–103. 10.1111/j.1365-2249.2004.02415.x 15030519 PMC1808997

[21] Prompetchara E Ketloy C Palaga T Immune responses in COVID-19 and potential vaccines: Lessons learned from SARS and MERS epidemic. Asian Pac J Allergy Immunol (2020) 38:1–9. 10.12932/AP-200220-0772 32105090

[22] Lamers D Famulla S Wronkowitz N Hartwig S Lehr S Ouwens DM Dipeptidyl peptidase 4 is a novel adipokine potentially linking obesity to the metabolic syndrome. Diabetes (2011) 60:1917–25. 10.2337/db10-1707 PMC3121429 21593202

[23] Sell H Blüher M Klöting N Schlich R Willems M Ruppe F Adipose dipeptidyl peptidase-4 and obesity: correlation with insulin resistance and depot-specific release from adipose tissue in vivo and in vitro. Diabetes Care (2013) 36:4083–90. 10.2337/dc13-0496 PMC3836153 24130353

[24] Karin N Razon H Chemokines beyond chemo-attraction: CXCL10 and its significant role in cancer and autoimmunity. Cytokine (2018) 109:24–8. 10.1016/j.cyto.2018.02.012 29449068

[25] Jia X Yin C Lu S Chen Y Liu Q Bai J Two things about COVID-19 might need attention. Preprints (2020). 10.20944/preprints202002.0315.v1

[26] Wan Y Shang J Graham R Baric RS Li F Receptor recognition by the novel coronavirus from Wuhan: an analysis based on decade-long structural studies of SARS coronavirus. J Virol (2020) 94(7):e00127-20. 10.1128/JVI.00127-20 31996437 PMC7081895

[27] Lu R Zhao X Li J Niu P Yang B Wu H Genomic characterisation and epidemiology of 2019 novel coronavirus: implications for virus origins and receptor binding. Lancet (2020) 395:565–74. 10.1016/S0140-6736(20)30251-8 PMC7159086 32007145

[28] Ohnuma K Hatano R Morimoto C DPP4 in anti-tumor immunity: going beyond the enzyme. Nat Immunol (2015) 16:791–2. 10.1038/ni.3210 26194276

[29] Samad A Jafar T Rafi JH Identification of angiotensin-converting enzyme 2 (ACE2) protein as the potential biomarker in SARS-CoV-2 infection-related lung cancer using computational analyses. Genomics (2020) 112:4912–23. 10.1016/j.ygeno.2020.09.002 PMC7831469 32916258

[30] Chan JF-W Yuan S Kok K-H To KK-W Chu H Yang J A familial cluster of pneumonia associated with the 2019 novel coronavirus indicating person-to-person transmission: a study of a family cluster. Lancet (2020) 395:514–23. 10.1016/S0140-6736(20)30154-9 PMC7159286 31986261

[31] Shin H-S Kim Y Kim G Lee JY Jeong I Joh J-S Immune responses to Middle East respiratory syndrome coronavirus during the acute and convalescent phases of human infection. Clin Infect Dis (2019) 68:984–92. 10.1093/cid/ciy595 PMC7108191 30060038

[32] Zhao J Zhao J Mangalam AK Channappanavar R Fett C Meyerholz DK Airway memory CD4+ T cells mediate protective immunity against emerging respiratory coronaviruses. Immunity (2016) 44:1379–91. 10.1016/j.immuni.2016.05.006 PMC4917442 27287409

[33] Drucker DJ Nauck MA The incretin system: glucagon-like peptide-1 receptor agonists and dipeptidyl peptidase-4 inhibitors in type 2 diabetes. Lancet (2006) 368:1696–705. 10.1016/S0140-6736(06)69705-5 17098089

[34] Nargis T Chakrabarti P Significance of circulatory DPP4 activity in metabolic diseases. IUBMB Life (2018) 70:112–9. 10.1002/iub.1709 29331088

[35] Nishina S Yamauchi A Kawaguchi T Kaku K Goto M Sasaki K Dipeptidyl peptidase 4 inhibitors reduce hepatocellular carcinoma by activating lymphocyte chemotaxis in mice. Cell Mol Gastroenterol Hepatol (2019) 7:115–34. 10.1016/j.jcmgh.2018.08.008 PMC6260362 30510994

[36] Hollande C Boussier J Ziai J Nozawa T Bondet V Phung W Inhibition of the dipeptidyl peptidase DPP4 (CD26) reveals IL-33-dependent eosinophil-mediated control of tumor growth. Nat Immunol (2019) 20:257–64. 10.1038/s41590-019-0321-5 30778250

[37] Solerte SB Di Sabatino A Galli M Fiorina P Dipeptidyl peptidase-4 (DPP4) inhibition in COVID-19. Acta Diabetol (2020) 1:61–5. 10.1007/s00592-020-01539-z PMC7275134 32506195

[38] Dalan R Is DPP4 inhibition a comrade or adversary in COVID-19 infection. Diabetes Res Clin Pract (2020) 164:108216. 10.1016/j.diabres.2020.108216 32416120 PMC7235575

