# Supplementary material for: Landscape Profiling Analysis of DPP4 in Malignancies: Therapeutic Implication for Tumor Patients With Coronavirus Disease 2019
Source: Front Oncol. 2021 Feb 4;11:624899. doi: 10.3389/fonc.2021.624899 (PMC7890191; doi:10.3389/fonc.2021.624899)
Supplement: Supplementary file 1 [file DataSheet_1.pdf]

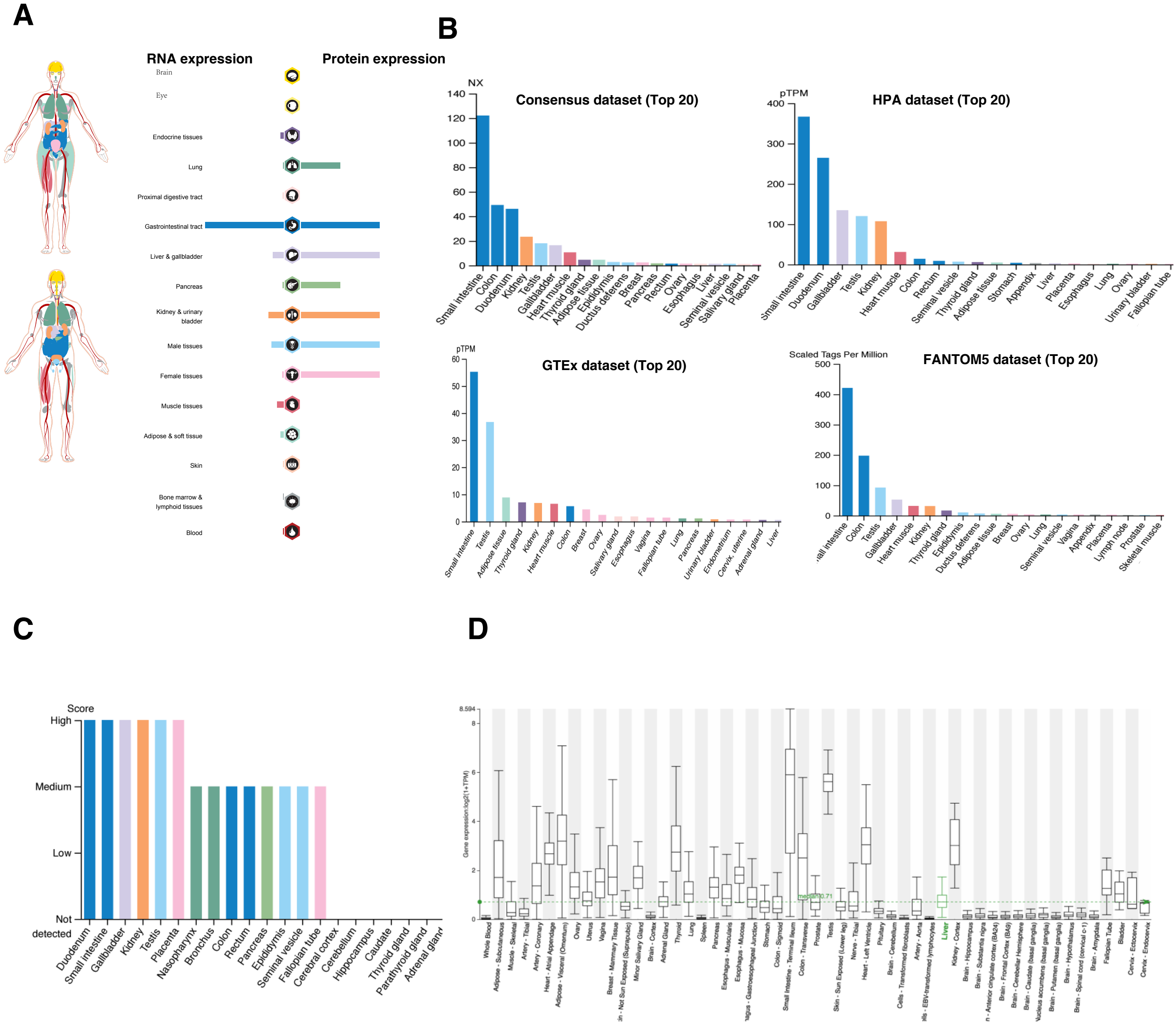

Supplementary Figure 1 Expression of ACE2 in human tissues and organs. (A) mRNA and protein expression profiles of ACE2 in all tissues and organs analyzed in the Tissue Atlas database; **(B)** Top 20 tissues or organs with ACE2 mRNA expression in four databases (Consensus dataset, Fantom5 dataset, GTEx dataset, and HPA dataset); **(C)** TOP20 tissues or organs with ACE2 protein expression in related database. **(D)** ACE2 mRNA expression in various tissues analyzed in the HCCDB database. ACE2, Angiotensin-converting enzyme 2.

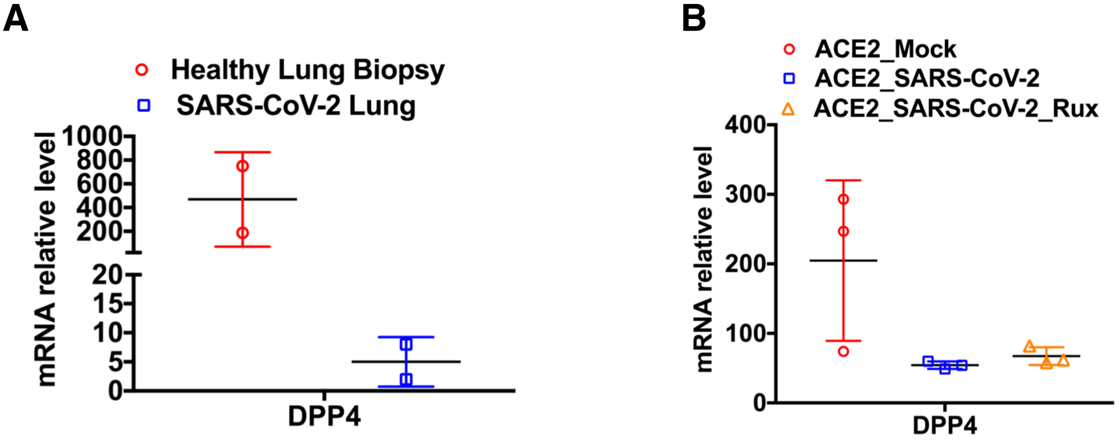

Supplementary Figure 2 DPP4 expression in SARS-CoV-2 infected lung tissue and cell lines. (A) mRNA level of DPP4 in lung of patient infected with SARS-CoV-2 (n=2). **(B)** The mRNA expression level of DPP4 in A549 cell line transfected with ACE2 promoter and then exposed to mock, SARS-CoV-2 (5×10^4 PFU) or SARS-CoV-2 (5×10^4 PFU) plus Ruxolitinib (500 nM) for 24 hours (n=3). Data are expressed as mean ± standard deviation.

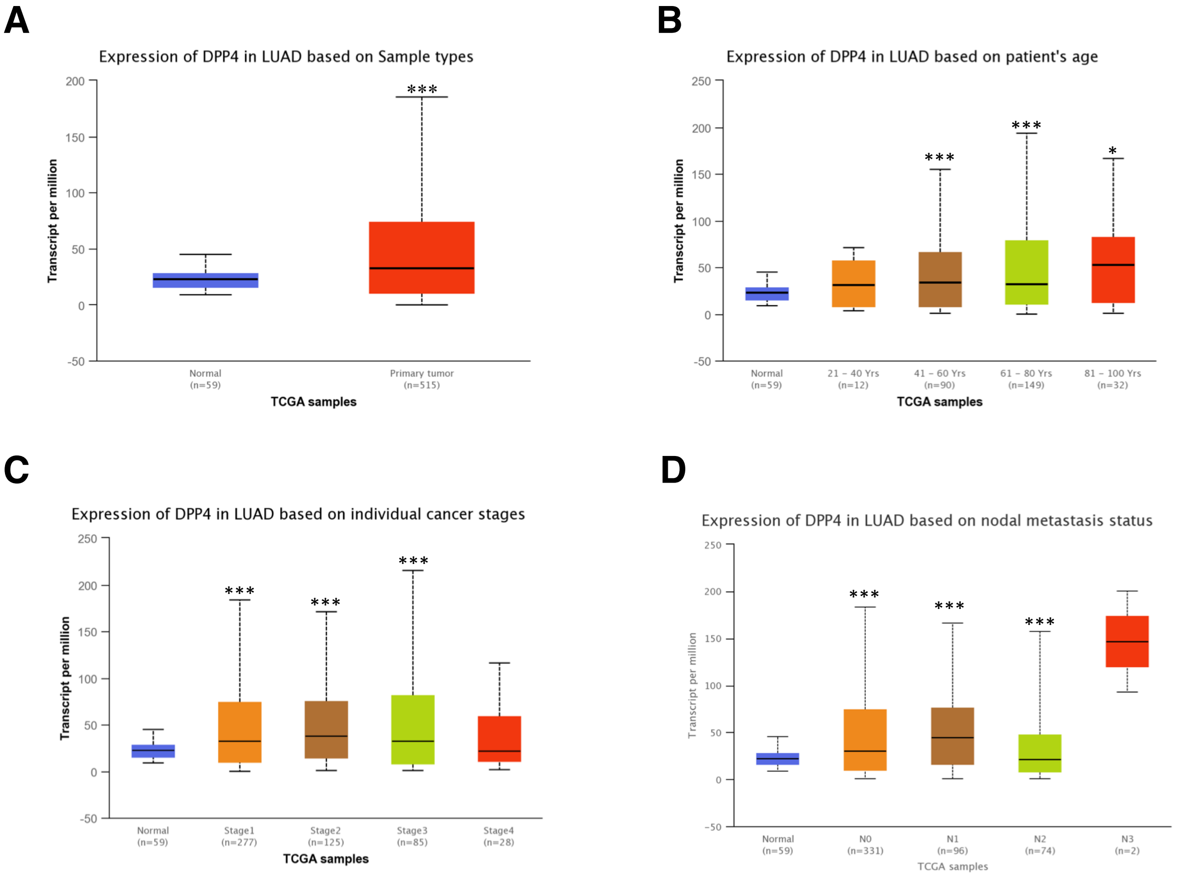

Supplementary Figure 3 Expression Profile of DPP4 in LUAD. (A-D) The expression of DPP4 in LUAD based on **(A)** sample types, **(B)** patient’s age, **(C)** individual cancer stages， and **(D)** nodal metastasis status. DPP4, dipeptidyl peptidase 4. LUAD, Lung adenocarcinoma, LUSC, Lung squamous cell carcinoma. N0, No regional lymph node metastasis; N1, Metastases in 1 to 3 axillary lymph nodes;N2, Metastases in 4 to 9 axillary lymph nodes;N3, Metastases in 10 or more axillary lymph nodes.

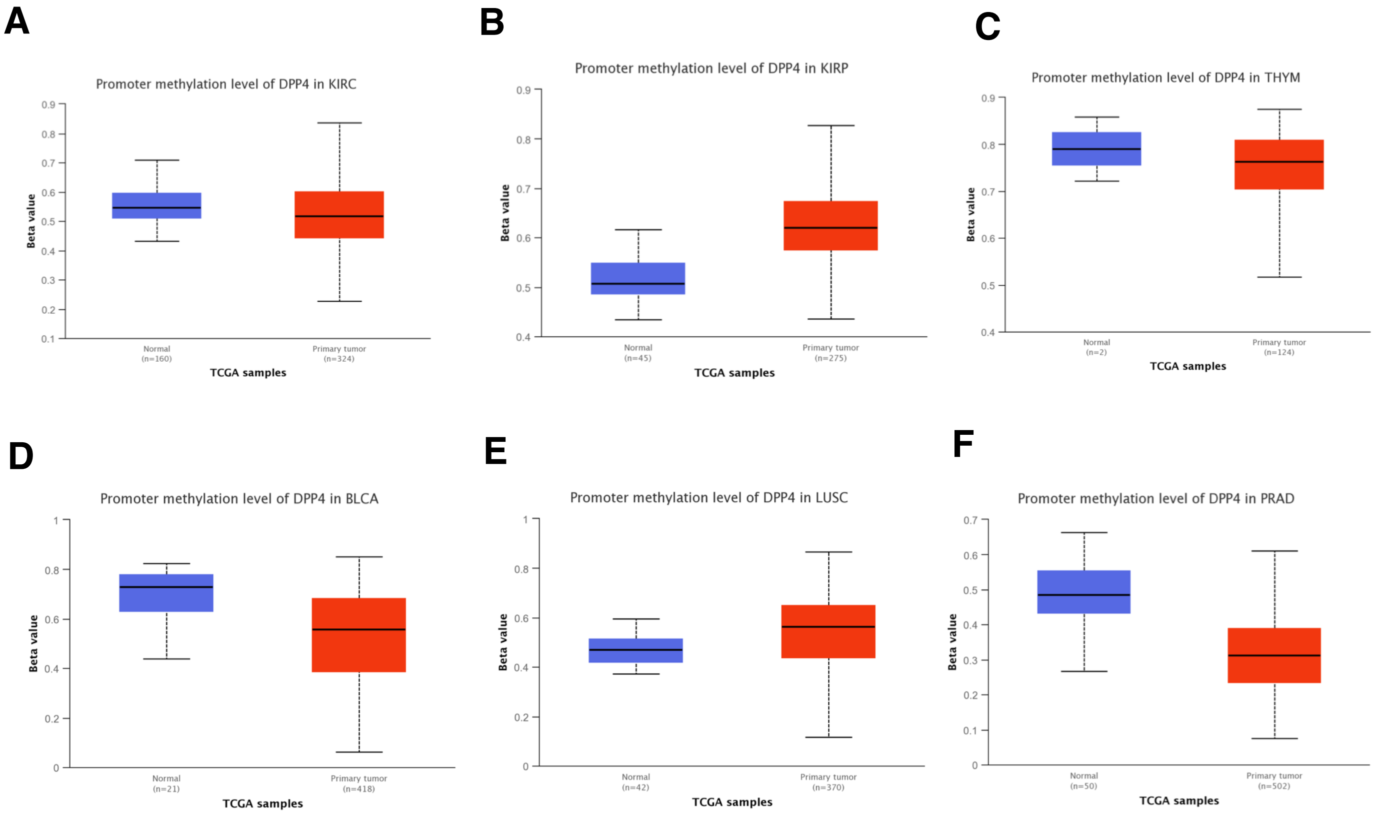

Supplementary Figure 4 The promoter methylation level of DPP4 in different types of cancer. (A-F) The promoter methylation level of DPP4 in **(A)** KIRC, **(B)** KIRP, **(C)** THYM, **(D)** BLCA, **(E)** LUSC, **(F)** PRAD. KIRC, kidney renal clear cell carcinoma; KIRP, kidney renal papillary cell carcinoma; THYM, thymoma; BLAC, bladder urothelial carcinoma; LUSC, Lung squamous cell carcinoma; PRAD, prostate adenocarcinoma.

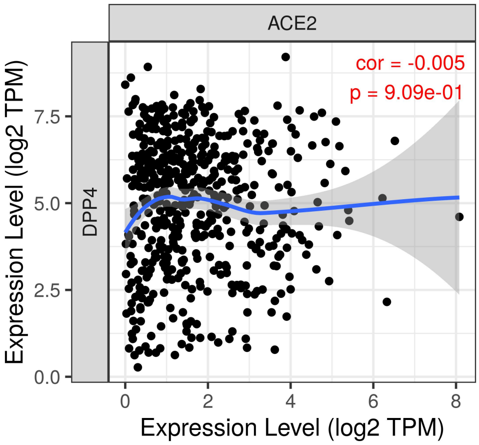

Supplementary Figure 5. Correlation of DPP4 and ACE2 in lung squamous cell carcinoma (LUSC) analyzed with TIMER. Scatterplots of correlations between DPP4 expression and gene markers of ACE2 in LUSC. DPP4 was used for the y-axis with gene symbols and on the x-axis, related marker genes are represented as gene symbols. The gene expression level was displayed using log2 RSEM. DPP4, Dipeptidyl-peptidase 4; ACE2, Angiotensin-converting enzyme 2.
