# Supplementary material for: Landscape Profiling Analysis of DPP4 in Malignancies: Therapeutic Implication for Tumor Patients With Coronavirus Disease 2019
Source: Front Oncol. 2021 Feb 4;11:624899. doi: 10.3389/fonc.2021.624899 (PMC7890191; doi:10.3389/fonc.2021.624899)
Supplement: Supplementary file 2 [file Table_1.docx]

**Supplementary table 1 The efficiency of DPP4 expression in the survival of pan-cancer patients.**

| cancer | infiltrates | z_score | p |
| --- | --- | --- | --- |
| ACC (n=79) | DPP4 | 2.047142556 | 0.04064409 |
| BLCA (n=408) | DPP4 | 0.999057498 | 0.317766838 |
| BRCA (n=1100) | DPP4 | 1.407958817 | 0.159143265 |
| BRCA-Basal (n=191) | DPP4 | -0.31502898 | 0.752739651 |
| BRCA-Her2 (n=82) | DPP4 | -1.627978373 | 0.103529472 |
| BRCA-LumA (n=568) | DPP4 | 1.655859057 | 0.097750365 |
| BRCA-LumB (n=219) | DPP4 | 1.592858738 | 0.111191885 |
| CESC (n=306) | DPP4 | 1.288984643 | 0.197403426 |
| CHOL (n=36) | DPP4 | 0.331202833 | 0.74049128 |
| COAD (n=458) | DPP4 | 0.706234869 | 0.480042089 |
| DLBC (n=48) | DPP4 | 1.875367485 | 0.060742185 |
| ESCA (n=185) | DPP4 | 1.036475993 | 0.299980134 |
| GBM (n=153) | DPP4 | -0.152358736 | 0.878904001 |
| HNSC (n=522) | DPP4 | 0.125666958 | 0.899995558 |
| HNSC-HPV- (n=422) | DPP4 | -0.594108787 | 0.552439354 |
| HNSC-HPV+ (n=98) | DPP4 | 1.615139081 | 0.106280579 |
| KICH (n=66) | DPP4 | 0.623495363 | 0.532959048 |
| KIRC (n=533) | DPP4 | -5.162245798 | 2.44E-07 |
| KIRP (n=290) | DPP4 | -2.987857684 | 0.002809403 |
| LAML (n=173) | DPP4 | 2.234472636 | 0.025451991 |
| LGG (n=516) | DPP4 | 8.609548747 | 7.33E-18 |
| LIHC (n=371) | DPP4 | 0.028143103 | 0.977548016 |
| LUAD (n=515) | DPP4 | -2.004922368 | 0.044971347 |
| LUSC (n=501) | DPP4 | 2.103042397 | 0.035462064 |
| MESO (n=87) | DPP4 | -1.361593925 | 0.173326074 |
| OV (n=303) | DPP4 | 0.637815431 | 0.523593833 |
| PAAD (n=179) | DPP4 | -0.844497062 | 0.398391704 |
| PCPG (n=181) | DPP4 | -1.175921194 | 0.239626369 |
| PRAD (n=498) | DPP4 | 0.937247373 | 0.348631327 |
| READ (n=166) | DPP4 | 0.423645448 | 0.671824405 |
| SARC (n=260) | DPP4 | -0.677789407 | 0.497905229 |
| SKCM (n=471) | DPP4 | -1.21605336 | 0.223964596 |
| SKCM-Metastasis (n=368) | DPP4 | -1.049943031 | 0.293744306 |
| SKCM-Primary (n=103) | DPP4 | -0.385986996 | 0.6995063 |
| STAD (n=415) | DPP4 | 0.976043009 | 0.329043152 |
| TGCT (n=150) | DPP4 | 1.011387392 | 0.311831053 |
| THCA (n=509) | DPP4 | -2.837080854 | 0.004552808 |
| THYM (n=120) | DPP4 | -2.373081403 | 0.017640376 |
| UCEC (n=545) | DPP4 | 0.473887249 | 0.635580313 |
| UCS (n=57) | DPP4 | 0.510978459 | 0.60936614 |
| UVM (n=80) | DPP4 | 0.837212505 | 0.402473129 |

Z score: Spearman’s ρ>0, p<0.05, positive correlation; ρ<0，p<0.05, negative correlation.
