# Supplementary material for: Landscape Profiling Analysis of DPP4 in Malignancies: Therapeutic Implication for Tumor Patients With Coronavirus Disease 2019
Source: Front Oncol. 2021 Feb 4;11:624899. doi: 10.3389/fonc.2021.624899 (PMC7890191; doi:10.3389/fonc.2021.624899)
Supplement: Supplementary file 3 [file Table_2.docx]

**Supplementary Table 2. Abbreviations for various cancers listed in Figure 5.**

| Cancer | Cancer Full Name |
| --- | --- |
| ACC | Adenoid cystic carcinoma |
| BLCA | Bladder Urothelial Carcinoma |
| BRCA | Breast Invasive Carcinoma |
| CESC | cervical squamous cell carcinoma and endocervical adenocarcinoma |
| [CHOL](http://starbase.sysu.edu.cn/panGeneDiffExp.php) | Cholangiocarcinoma |
| COAD | Colon Adenocarcinoma |
| DLBC | Diffuse large B-cell lymphoma |
| ESCA | Esophageal Carcinoma |
| GBM | Glioblastoma multiforme |
| HNSC | Head and Neck Squamous Cell Carcinoma |
| KICH | Kidney Chromophobe |
| KIRC | Kidney Renal Clear Cell Carcinoma |
| KIRP | Kidney Renal Papillary Cell Carcinoma |
| Cancer | **Cancer Full Name** |
| LGG | Low Grade Glioma |
| LIHC | Liver Hepatocellular Carcinoma |
| LUAD | Lung Adenocarcinoma |
| LUSC | Lung Squamous Cell Carcinoma |
| MESO | Mesothelioma |
| OV | Ovarian Serous Cystadenocarcinoma |
| PAAD | Pancreatic Adenocarcinoma |
| PCPG | Pheochromocytoma/paraganglioma |
| PRAD | Prostate Adenocarcinoma |
| READ | Rectum Adenocarcinoma |
| SARC | Sarcoma carcinoma |
| SKCM | Skin cutaneous melanoma |
| STAD | Stomach Adenocarcinoma |
| TGCT | Testicular germ cell tumors |
| THCA | Thyroid Carcinoma |
| THYM | Thymoma |
| UCS | Uterine carcinosarcoma |
| UVM | Uveal Melanoma |
| UCEC | Uterine Corpus Endometrial Carcinoma |
